# Executive attention impairment in first-episode schizophrenia

**DOI:** 10.1186/1471-244X-12-154

**Published:** 2012-09-22

**Authors:** Gricel Orellana, Andrea Slachevsky, Marcela Peña

**Affiliations:** 1 Departamento de Psiquiatría Oriente, Facultad de Medicina, Universidad de Chile, Av. Salvador 486, Providencia, Santiago, Chile; 2 Centro de Investigación Avanzada en Educación, Universidad de Chile, Periodista Jose Carrasco Tapia 75, Santiago, Chile; 3 Departamento de Neurología Oriente, Facultad de Medicina, Universidad de Chile, Av. Salvador 364, Providencia, Santiago, Chile; 4 Servicio de Neurología, Hospital del Salvador, Santiago, Chile, Av. Salvador 364, Providencia, Santiago, Chile; 5 Escuela de Psicología, Pontificia Universidad Católica de Chile, Av. Vicuña Mackenna 4860, Macul, Santiago, Chile; 6 Scuola Internazionale Superiore Studi Avanzati, Via Bonomea 265, Trieste, 34136, Italy

**Keywords:** Schizophrenia, First-episode, Attention, ANT, Executive, Cognitive

## Abstract

**Background:**

We compared the attention abilities of a group of first-episode schizophrenia (FES) patients and a group of healthy participants using the Attention Network Test (ANT), a standard procedure that estimates the functional state of three neural networks controlling the efficiency of three different attentional behaviors, i.e., alerting (achieving and maintaining a state of high sensitivity to incoming stimuli), orienting (ability to select information from sensory input), and executive attention (mechanisms for resolving conflict among thoughts, feelings, and actions).

**Methods:**

We evaluated 22 FES patients from 17 to 29 years of age with a recent history of a single psychotic episode treated only with atypical neuroleptics, and 20 healthy persons matched with FES patients by sex, age, and educational level as the control group. Attention was estimated using the ANT in which participants indicate whether a central horizontal arrow is pointing to the left or the right. The central arrow may be preceded by spatial or temporal cues denoting where and when the arrow will appear, and may be flanked by other arrows (hereafter, flankers) pointing in the same or the opposite direction.

**Results:**

The efficiency of the alerting, orienting, and executive networks was estimated by measuring how reaction time was influenced by congruency between temporal, spatial, and flanker cues. We found that the control group only demonstrated significantly greater attention efficiency than FES patients in the executive attention network.

**Conclusions:**

FES patients are impaired in executive attention but not in alerting or orienting attention, suggesting that executive attention deficit may be a primary impairment during the progression of the disease.

## Background

Schizophrenia is a mental illness affecting 1% of the world population and has severe deleterious effects on quality of life; mainly because symptoms begin at an early age and full recovery has not been achieved with current therapies [1]. Schizophrenia is characterized by multiple cognitive impairments including attention deficit [2]. Most psychosocial problems in schizophrenia are associated with cognitive deficiency [3-6].

The majority of studies on cognition in schizophrenia involve heterogeneous samples of adults suffering from chronic schizophrenia with long histories of somatic treatments including electroconvulsive therapy. Thus, the nature of neurocognitive dysfunction is potentially confounded by the effects of age, clinical symptoms, illness duration and severity, and/or treatment. Over the past 15 to 20 years, however, there has been a growing interest in the clinical and neurocognitive characteristics of the early phases of schizophrenia, an approach that has the potential to minimize many of the interpretive difficulties associated with studying chronically ill patients [7].

Attention deficits have been reported in schizophrenia from the earliest descriptions of the disease [8]. As one of the earliest clinical manifestations of schizophrenia, attention impairment may be a primary disorder in the neuropathology of schizophrenia [9,10]. Most data on attention deficit in schizophrenia have involved chronic patients and have found that these patients demonstrate impairment in all three attention networks (evaluated with the Attention Network Test; ANT). Yet, studies of chronic schizophrenia provide little information to shed light on which attention capacity is primarily impaired and which may explain the first manifestations of the disease.

Attention is not a single unitary system but a set of integrated processes that act on all levels of cognitive processing from sensory entry to motor exit [9,11]. As early as Bleuler’s (1911) articles, many authors have been led to consider attentional disorder as a basic manifestation of the development of the illness.

Attention in schizophrenia has been primarily studied with the Stroop test and the Continuous Performance Test (CPT). The Stroop test estimates how reaction time slows when a participant must deal with conflicting information. In the classic Stroop test, participants need to say, as fast as possible, the name of the color of words written in ink of the same or of a different color from that which is specified by the letters (e.g., the word “blue” written in green ink). Participants take longer to say the name of the color when the written words are different from the ink color. The Stroop test is presented on printed cards or on a computer screen. In the card version of this test, under conditions of conflict, patients suffering from chronic schizophrenia show slower reaction times and a higher error rate than control participants. Therefore, schizophrenic patients present an increase in degree of sensitivity to interference [12]. Studies carried out with the computer version of the Stroop test have shown that, when compared to healthy control participants, schizophrenic patients present a higher error rate in situations ranging from the neutral to the incongruent or conflicting condition [12]. Deficits in the execution of the CPT have been detected in chronic schizophrenia and in adolescent and adult patients during early stages of the illness, in nonpsychotic relatives of patients, and in offspring at risk of inheriting the illness from their schizophrenic parents [13]. The totality of these studies suggest that the sustained attention deficit, as measured by the CPT, is stable during the course of the illness, does not improve with antipsychotic treatment, and has a strong genetic component. CPT deficits would appear to be specific to schizophrenia; they are not found in depression and in adolescents that are at risk of suffering affective illnesses [13].

Current models have shown that attention is not a unitary function but the result of three different attentional networks, i.e., alerting, orienting, and executive function [14], that can be independently evaluated using the ANT [15-17]. Alerting is manifested by achieving and maintaining the alert state, orienting by the ability to direct attention to sensory events, and executive attention by efficient control of thoughts, actions, and feelings. The ANT has been widely validated across a number of cultures, ages, and morbid entities. Some recent studies exploring attention in chronic schizophrenia with the ANT have revealed contrasting results: One study reported impairment in orientation and executive attention [18], whereas others referred exclusively to a deficit in executive attention [19-21] or in alerting [22]. Still other studies have produced controversial results, reporting smaller conflict effect scores in individuals with chronic schizophrenia measured behaviorally [23] and by event-related potentials [24]. Finally, another study observed that positive syndrome patients showed less efficiency than healthy controls in the orientation network [25]. The discrepancy reported by these studies may be due to the heterogeneity of symptoms and syndromes in chronic schizophrenic patients. For example, the sample described by Gooding et al. [19] consisted of chronic schizophrenia–spectrum patients including chronic schizophrenia and schizoaffective disorder. However, the inconsistency can also be a consequence of differences in the progression and treatment of the disease. Studying patients with first-episode schizophrenia (FES) provides the advantage of being able to control the different factors described above. A consistent impairment in attention exists in FES [7]; nevertheless, to the best of our knowledge, attention networks in FES patients have not been evaluated with the ANT. The aim of our study was to establish which attentional network is impaired in schizophrenia during early stages of the disease treated only with one atypical antipsychotic.

Since schizophrenia is highly related to dopamine and prefrontal dysfunction in its earliest stages, we may expect that FES patients exhibit exclusive reduction in executive attention [26,27]. Indeed, in monkeys, alerting is more influenced by norepinephrine [28] and orienting by acetylcholine [29-31]. We thus hypothesize that, contrary to patients with chronic schizophrenia, FES patients will only show a primary deficit in executive attention.

## Methods

### Participants

Twenty-two FES participants took part in this study. FES patients were recruited between 2004 and 2009 from Psychiatry Services of Hospital Barros Luco Trudeau and Hospital Salvador in Santiago, Chile. The 22 FES patients met the clinical and DSM-IV-TR criteria for schizophrenia in the Structured Clinical Interview for DSM-IV Axis I Disorders (SCID-I). Nineteen FES patients were paranoid, two disorganized, and one catatonic. They were evaluated during a period ranging from 1 to 36 months after clinical diagnosis with no history of other episodes prior to or after diagnosis. After a psychotic episode, FES patients were rated on the Positive and Negative Syndrome Scales (PANSS). Their scores on the Positive Syndrome Scale ranged from 7 to 13 (mean = 8.5 ± 2.0) and on the Negative Syndrome Scale ranged from 7 to 28 (mean = 16.8 ± 6.2), corresponding to mild schizophrenia. During the evaluations of our study, FES patients were clinically stable, receiving a single atypical antipsychotic medication, i.e., Risperidone in 14 FES patients (dose: 4.5-9 mg/day), Olanzapine in three FES patients (dose: 20–30 mg/day), Clozapine in four FES patients (dose: 125–550 mg/day), and Quetiapine in one FES patient (dose: 250 mg/day). Computed cerebral tomography of 20 patients showed no brain abnormalities. Two patients refused to participate. FES patients showed no significant extrapyramidal symptoms upon evaluation with the Extrapyramidal Symptom Rating Scale (ranging from 0 to 10 with a maximum score of 175) and reported no visual or hearing problems. Every participant verbally confirmed that they correctly understood the procedure and the tasks. All evaluations were carried out in the University of Chile, Department of Psychiatry. Twenty healthy control participants, matched with FES patients by age, gender, and years of education, were also included in the study.

For both FES and control participants, exclusion criteria included history of: (a) brain disease other than schizophrenia, (b) mental retardation, (c) substance abuse, or (d) electroconvulsive therapy. The study was approved by the regional ethics committee for biomedical research of the University of Chile, Faculty of Medicine. Before participating in any evaluation, all participants were invited to participate by a psychiatrist who explained the study’s goal and steps among other study-related information, and gave a copy of the written informed consent. All participants signed the written informed consent before starting the first evaluation.

### Procedure

FES participants were evaluated in three consecutive sessions. In the first session, the SCID-I was used to verify the clinical diagnosis and the PANSS was administered. In the second session, global cognition was estimated with the Dementia Rating Scale (DRS) and Raven’s Progressive Matrices (RPM), two standard scales evaluating cognitive efficiency in adults. In the third session, FES participants underwent the ANT.

Participants of the control group were evaluated in only two sessions. In the first session, the absence of mental disorder was verified using the SCID-I, then global cognition was estimated with the DRS and RPM. In the second session, control participants underwent the ANT. The DRS and RPM tests were administered to FES and control groups to corroborate the differences in global cognition between both groups and to determine their correlation with the efficiency of attention networks.

### Cognitive assessments

#### Global cognition

The DRS global score estimates combined cognitive functioning with the following components: attention, initiation-perseveration, construction, conceptualization, and memory domains [32]. Some DRS items also assess overall attentional capacity [33], which can be calculated by adding the scores of the following untimed tasks to produce what we will refer to as the DRS attention score: digit span forward and backward, motor response to single commands, visual scanning, word list reading, and visual figure matching. The RPM raw score estimates reasoning by asking participants to determine the missing segment required to complete a larger pattern from a series of geometric arrangements that become progressively more difficult to solve [34].

#### The ANT

**Figure 1:**
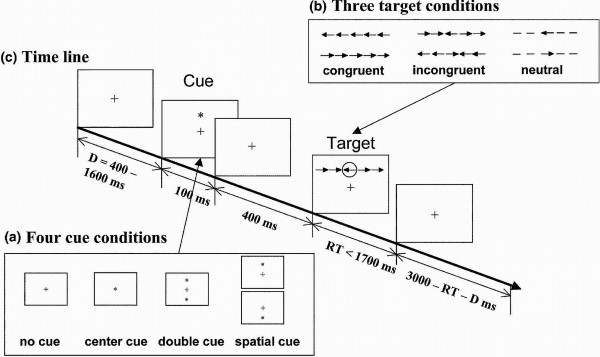
**ANT. a**) There are four different cue types: No-cue (i.e., no cues displayed), Center-cue (i.e., cue displayed in the center of the screen), Double-cue (i.e., two horizontally centered cues simultaneously displayed), and Spatial-cue (i.e., cue appears in the same place where the target will subsequently appear); **b**) Flankers are lines (neutral condition) or arrows pointing in the same (congruent condition) or opposite (incongruent condition) direction as that of the central target arrow; **c**) Timeline of the trials. (Adapted from “A symbolic model of human attentional networks" by H. Wang, J. Fan and T.R. Johnson, 2004, Cognitive Systems Research *5*, p. 121. Reprinted with permission).

The ANT is a standard task described in detail in Fan et al., 2002 (Figure 1). In the ANT, stimuli are visually presented on a screen and participants are instructed to respond, as quickly and accurately as possible, by pressing either a left or a right button indicating the direction of a centered target arrow (pointing leftward or rightward), irrespective of flanking and cue clues. Flankers are lines (neutral condition) or arrows pointing in the same (congruent condition) or opposite (incongruent condition) direction as that of the central target arrow. Cues are asterisks that can be present or absent. When cues are present, they appear 400 ms before the stimuli and provide information about where and when the subsequent stimuli will appear. There are four different cue types: No-cue (i.e., no cues displayed), Center-cue (i.e., cue displayed in the center of the screen), Double-cue (i.e., two horizontally centered cues simultaneously displayed, one 1.5 cm above and one 1.5 cm below a central fixation point), and Spatial-cue (i.e., cue appears in the same place where the target will subsequently appear). To estimate performance of the attention networks, the four cue types and three flanker types generate 12 experimental conditions: No-cue congruent, No-cue incongruent, No-cue neutral, Central-cue congruent, Central-cue incongruent, Central-cue neutral, Double-cue congruent, Double-cue incongruent, Double-cue neutral, Spatial-cue congruent, Spatial-cue incongruent, and Spatial-cue neutral. The ANT was presented in four blocks: one full-feedback practice block of 24 trials followed by three experimental blocks without feedback of 96 trials each. The 96 trials resulted from the combination of four cues (No-cue, Central-cue, Double-cue, and Spatial-cue), three congruency types (Congruent, Incongruent, and Neutral), two directions (Left and Right), and two locations (Up and Down) with each combination presented twice. Trials from different experimental conditions were randomly presented. The practice block took 2 minutes while each experimental block lasted nearly 5 minutes. A small cross served as a central fixation point. Participants were instructed to direct their gaze toward the fixation point throughout the task. We computed the mean accuracy and mean reaction time (RT) of the trials with correct responses for each experimental condition. Then, we estimated the efficiency of the alert, orientation, and executive attention networks using a series of RT subtractions between different experimental conditions: a) Alert network = No-cue RT – Double-cue RT; b) Orientation network = Center-cue RT – Spatial-cue RT; and c) Executive network = Incongruent RT – Congruent RT [15].

### Statistical analysis

#### Analysis of demographic, clinical, and global cognition data

Gender, age, years of education, and DRS and RPM scores of FES and control groups were submitted to a *t* test for independent samples (alpha = .05; two-tailed). Moreover, to measure global attention with a clinical tool, we submitted the DRS subscore estimating attention in FES and control groups to a one-way ANOVA.

#### ANT analysis

The mean accuracy and RT in each experimental condition were submitted to two separate repeated-measures ANOVA with Target direction (Left & Right), Flanker type (Neutral, Congruent, & Incongruent), and Cue type (No, Central, Double, & Spatial cues) as within-subjects factors, and Group (FES & Control) as the between-subjects factor. The mean efficiency for alert, orientation and executive attention networks was submitted to a repeated-measures ANOVA with Network (Alert, Orientation, & Executive) as the within-subjects factor and Group (FES & Control) as the between-subjects factor. The Greenhouse-Geisser correction was applied in all comparisons.

#### Correlations

We estimated the independence of attention networks by submitting the mean efficiency between all possible pairs of attention networks in FES and control participants to a Spearman correlation analysis. Moreover, we explored the correlation between attention network efficiency and global cognition by evaluating the mean DRS and RPM scores against the mean efficiency of each attention network in FES and control participants in another Spearman correlation analysis.

## Results

### Demographic and clinical data

A comparison of demographic and clinical data between the two groups of participants is illustrated in Table 1. There were no significant differences between groups according to gender (9 versus 8 women in the FES and control groups respectively; *t*_(41)_ *=* 0.059, *p =* .954), age (*t*_(41)_ *= −*1.463, *p =* .151), or years of education (*t*_(41)_ *=* 1.224, *p =* .228).

**Table 1:** Demographic and clinical data of the FES and control groups

	**FES patients (*n*** **= 22)**		**Control group (*n*** **= 20)**	
	**Mean**	**Standard deviation**	**Mean**	**Standard deviation**
Age (years)	21.8	3.5	20.3	3.2
Education level (years)	12.1	2.4	13	2.2
DRS*	131.7	8.3	141.7	1.6
RPM*	34.9	11.4	55.6	3.1
PANSS POSITIVE	8.5	2.0	NA	NA
PANSS NEGATIVE	16.8	6.2	NA	NA
PANSS PSYCHOP.	26.36	5.90	NA	NA
PANSS TOTAL	51.66	11.48	NA	NA

* *p* < .05.

### Global cognition

Results on global cognition are described in Table 1. Global cognition was significantly lower in FES participants compared to control participants in both the DRS (*t*_(41)_ = 5.5, *p* < .001) and RPM (*t*_(41)_ = 8.2, *p* < .001). Consistent with attention deficiency in FES, the mean overall attentional capacity estimated by the DRS’s attention subscore was also significantly lower for FES participants than controls (*F*_(1,36)_ = 32.7, *p* < .001).

### The ANT

#### Accuracy

**Figure 2:**
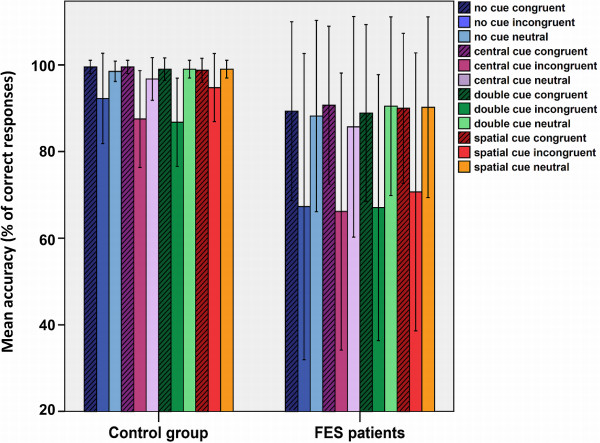
**Mean accuracy for each experimental condition and group.** We plotted the mean accuracy for each experimental condition for the FES and control groups. Vertical lines indicate one standard deviation of the mean.

Accuracy results are illustrated in Figure 2. We found a main effect of Flanker type (*F*_(1, 42)_ = 24.8, *p* < .001), a main effect of Cue type (*F*_(3,109)_ = 4.8, *p* < .005), and two significant interactions: Flanker type X Group (*F*_(1,42)_ = 4.7, *p* < .03) and Cue type X Flanker type (*F*_(4,171)_ = 4.1, *p* < .003). A post-hoc analysis revealed that: a) The mean accuracy was significantly lower for the Incongruent condition compared to Congruent (*p* < .001) and Neutral (*p* < .001) conditions; b) The mean accuracy was significantly higher for the Spatial-cue condition compared to the Central-cue (*p* < .001) and Double-cue (*p* < .006) conditions; c) FES participants presented a significantly lower mean accuracy than the control group only in the Incongruent condition (*p* < .04); and d) only in the Incongruent condition was the mean accuracy significantly higher for Spatial-cue trials compared to Central-cue (*p* < .002) and Double-cue (*p* < .001) trials. No significant main effects or interactions were observed for Target Direction.

#### RT

**Figure 3:**
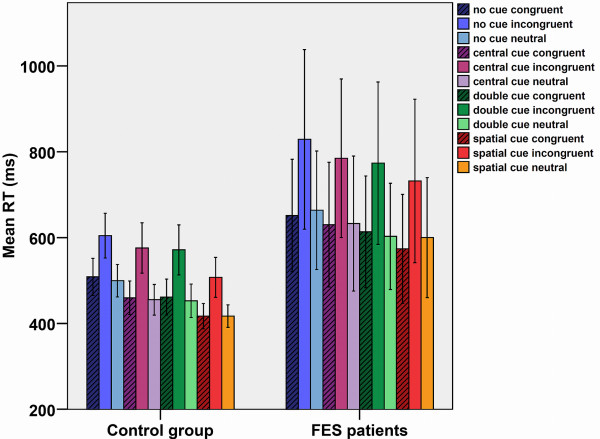
**Mean reaction time for each experimental condition and group.** We plotted the mean RT for each experimental condition for the FES and control groups. Vertical lines indicate one standard deviation of the mean.

Results on RTs are plotted in Figure 3. We observed a main effect of Cue type (*F*_(2,94)_ = 51.7, *p* < .001), a main effect of Flanker type (*F*_(1,58)_ = 119.9, *p* < .001), and a significant interaction of Flanker type X Group (*F*_(1,58)_ = 4.0, *p* < .04). A post-hoc analysis revealed that: a) The mean RT was significantly higher for the Incongruent condition than for the Congruent (*p* < .001) and Neutral (*p* < .009) conditions; b) The mean RT was significantly higher for the No-cue condition than for the Central-cue (*p* < .001), Double-cue (*p* < .001), and Spatial-cue (*p* < .001) conditions; and c) FES participants showed a significantly higher mean RT than the control group only in the Incongruent condition (*p* < .007). Neither significant main effects nor interactions were observed for Target Direction.

### Efficiency of attention networks

**Figure 4:**
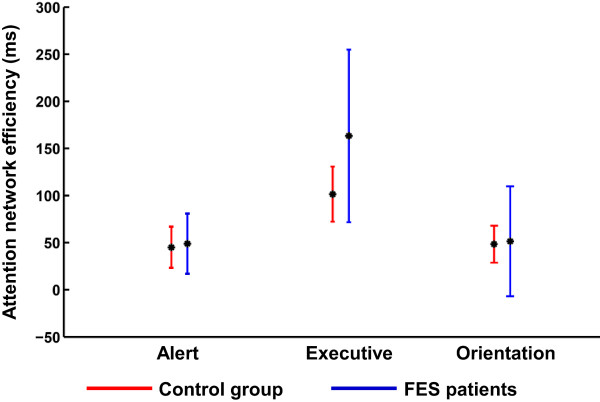
**Mean efficiency of the attention networks.** The mean efficiency of the alerting, orienting, and executive attention networks for each group are plotted. Black dots indicate the mean efficiency and vertical bars indicate one standard error of the mean.

Results on the efficiency of the attention networks are presented in Figure 4. A main effect of Network (*F*_(1,55)_ = 39.0, *p* < .001) and a significant interaction of Network X Group (*F*_(1,55)_ = 4.0, *p* < .04) were observed. Post-hoc analysis showed that the variation in the mean RT was significantly higher for the Executive network than for the Alert (*p* < .001) and Orientation (*p* < .001) networks. Moreover, the control group demonstrated significantly greater attention efficiency than the FES group only in the Executive attention network (*p* < .009). Neither significant main effects nor interactions were observed for Target Direction.

### Independence of attention networks

No significant correlations were found between any pair of attentional networks.

### Correlation between attention and global cognition

When we compared the entire sample (i.e., FES and control participants), the RPM raw score correlated negatively with the efficiency of the Executive network (Spearman’s *rho* = −.32, *p* < .04). However, when we restricted the comparison to each group, the correlation was not significant.

## Discussion

To the best of our knowledge, our study is the first to explore the efficiency of alerting, orienting, and executive attention in FES patients using the ANT. In our sample, FES participants were all being treated with atypical antipsychotics and no other drugs that affect cognition, constituting a relatively homogenous group of patients with schizophrenia. Our results showed that FES participants have impairment in executive attention, even when clinical manifestations of the disease are controlled by antipsychotic drugs. The efficiency of executive attention in FES participants was lower than observed for control participants. In contrast, alerting and orienting attention were not significantly impaired in FES participants. Our results are consistent with previous studies in chronic patients using the ANT [18,19,21]. However, studies focusing solely on male patients [23], classifying by gender [20], using event-related potentials [24], and concentrating on symptoms measured by the PANSS [25] did not report specific impairments in executive attention. This may be explained by the heterogeneity of symptoms, syndromes, and treatments in chronic schizophrenic patients, the level of nicotine that may improve attention in these patients [35], or the lack of evaluation of global cognition in some of these studies. Although our study does not endeavor to propose any causal relationships between initial executive attention damage and later difficulty with alerting or orienting, impairments in executive attention in the early stages of schizophrenia may play a crucial role in the progression of the disease.

The independence of the three attention networks evaluated with the ANT has been controversial. Although Fan et al. (2002), did not find significant correlations between the networks in their first report, they did observe interactions between them [15]. A recent study involving the analysis of 1,141 healthy persons (from different studies) [36] has shown that the level of correlation between the attention networks is influenced by experimental manipulations. The above-mentioned study identifies an interaction between the Cue and Flanker conditions in all studies, supporting the idea that attentional networks influence each other. In our study, we also found a significant Cue X Flanker interaction in both FES and control groups due to the significant improvement of performance in incongruent trials when spatial cues were present. These results did not demonstrate network-specific effects but allow for the possibility that initial impairment in executive attention may influence the functioning of alerting and/or orienting attentional networks in more advanced stages of the disease.

Similar to previous reports [18,37-40], the impairment in executive attention cannot be directly attributed to a global cognition deficiency in our study. Although the DRS total scores, DRS attention scores, and RPM scores were significantly lower for FES than for control participants, they did not significantly correlate to the efficiency of executive attention, accuracy, or RT for any condition for either group. The poorer performance in cognitive tasks observed in schizophrenia patients has also been associated with an overall cognitive slowdown, secondary to a reduction in motor and information processing speed [7,37,39,41], drowsiness, and extrapyramidal symptoms induced by drugs [42,43]. Our results could not be directly attributed to these factors because FES participants did not exhibit a significant decrease in RT for alerting and orienting networks, or any extrapyramidal problems.

Executive attention deficits have been described in several neuropsychiatric pathologies such as schizophrenia–spectrum and schizoaffective disorders [19], Alzheimer disease, 22q11 deletion syndrome, borderline personality disorder [44], post-traumatic stress disorder [45], obesity accompanied by severe psychiatric co-morbidity [46], and attentional deficit disorder [47]. These deficits may thus underpin a large range of self-regulation disorders [17]. Self-regulation refers to the ability to monitor and modulate the cognitive state, emotions, and behaviors to accomplish goal-directed tasks, and to adapt to specific cognitive and social demands [48,49]. Our results mesh well with this proposal in suggesting that early executive attention deficits in FES patients might explain their difficulties in exerting control over thoughts, feelings, and actions [50], and over some disexecutive behaviors [51]. Further studies with larger samples and applying other cognitive assessment batteries are necessary to pinpoint the exact role of executive attention in specific cognitive problems of FES patients. Our results, however, contribute with new data that can potentially facilitate a better understanding of the physiopathology, diagnosis, and rehabilitation of schizophrenia.

## Conclusion

In this study, we observed that FES patients are impaired in executive attention but not in alerting or orienting attention. Our findings suggest that executive attention deficit may be a primary impairment during the progression of the disease.

## Competing interests

There are no potential conflicts of interest to report for the authors or other contributors to this research.

## Authors’ contributions

All authors participated in the conception and design of the study, acquisition of the data, analysis and interpretation of the results, and drafting of the manuscript. Furthermore, all authors read, critically revised, and approved the final manuscript.

## Pre-publication history

The pre-publication history for this paper can be accessed here:

http://www.biomedcentral.com/1471-244X/12/154/prepub
